# On the Computational Power of Spiking Neural P Systems with Self-Organization

**DOI:** 10.1038/srep27624

**Published:** 2016-06-10

**Authors:** Xun Wang, Tao Song, Faming Gong, Pan Zheng

**Affiliations:** 1 College of Computer and Communication Engineering, China University of Petroleum, Qingdao 266580, Shandong, China; 2 Faculty of Engineering, Computing and Science, Swinburne University of Technology Sarawak Campus, Kuching, 93350, Malaysia

## Abstract

Neural-like computing models are versatile computing mechanisms in the field of artificial intelligence. Spiking neural P systems (SN P systems for short) are one of the recently developed spiking neural network models inspired by the way neurons communicate. The communications among neurons are essentially achieved by spikes, i. e. short electrical pulses. In terms of motivation, SN P systems fall into the third generation of neural network models. In this study, a novel variant of SN P systems, namely SN P systems with self-organization, is introduced, and the computational power of the system is investigated and evaluated. It is proved that SN P systems with self-organization are capable of computing and accept the family of sets of Turing computable natural numbers. Moreover, with 87 neurons the system can compute any Turing computable recursive function, thus achieves Turing universality. These results demonstrate promising initiatives to solve an open problem arisen by Gh Păun.

In the central nervous system, there are abundant amount of computational intelligence precipitated throughout millions of years of evolution. The computational intelligence has provided plenty of inspirations to construct powerful computing models and algorithms^1^,^2^,^3^. Neural-like computing models are a class of powerful models inspired by the way how neurons communicate. The communication among neurons is essentially achieved by spikes, i.e. short electrical pulses. The biological phenomenon has been intensively investigated in the field of neural computation^4^. Using different mathematic approaches to describe neural spiking behaviours, various neural-like computing models have been proposed, such as artificial neural networks^5^ and spiking neural networks^6^. In the field of membrane computing, a kind of distributed and parallel neural-like computation model, named spiking neural P systems (SN P systems), were proposed in 2006^7^. SN P systems are widely considered as a promising variant of the third generation of neural network models^8^.

Generally, an SN P system can be represented by a directed graph, where neurons are placed in nodes and the synapses are denoted using arcs. Every neuron can contain a number of spikes and a set of firing (or spiking) rules. Following the firing rules, a neuron can send information encoded in spikes to other neurons. Input neurons read spikes from the environment, and output neurons emit spikes into the environment. The computation result can be embodied in various ways. One of the common approaches is the time elapsed between the first two consecutive spikes sent into the environment^9^,^10^ and the total number of spikes emitted into the environment^11^,^12^,^13^.

For the past decade, there have been quite a few research efforts put forward to SN P systems. Notably, SN P systems can generate and accept the sets of Turing computable natural numbers^14^, generate the recursively enumerable languages^15^,^16^ and compute the sets of Turing computable functions^17^. Inspired by different biological phenomena and mathematical motivations, lots of variants of SN P systems have been proposed, such as SN P systems with anti-spikes^18^,^19^, SN P systems with weight^20^, SN P systems with astrocyte^21^, homogenous SN P systems^22^,^23^, SN P systems with threshold^24^, fuzzy SN P systems^25^,^26^, sequential SN P systems^27^, SN P systems with rules on synapses^28^, SN P systems with structural plasticity^29^. For applications, SN P systems are used to design logic gates, logic circuites^30^ and operating systems^31^, perform basic arithmetic operations^32^,^33^, solve combinatorial optimization problems^34^, diagnose fault of electric power systems^35^.

SN P systems are known as a class of neural-like computing models under the framework of membrane computing^36^. Spiking neural network (shortly named SNN) is a well known candidate of siking neural network models^37^, which incorporates the concept of time into their operating model, besides neuronal and synaptic state in general artificial neural networks, The neuron in SNN cannot fire at each propagation cycle, but only when a membrane potential reaches a specific value. When a neuron fires, it generates a signal which travels to other neurons which, in turn, increase or decrease their potentials in accordance with this signal. In SN P systems, spiking rules, denoted by formal production in grammar theory of formal languages, is used to describe the neuron's spiking behaviour, which determine the conditions of triggering spiking, the number of spikes consumed, and the number of spikes emitting to the neighboring neurons. The spikes from different neurons can be accumulated in the target neuron for further spiking. In terms of motivation of models, SN P systems also fall into the spiking neural network models, i.e., the third generation of neural network models.

Since SN P systems have more fundamental data structure (spike trains, i.e., binary strings), it performs well in achieving significant computation power with using a small number of units (neurons). It was proved by Gh Păun that 49 neurons are sufficient for SN P systems to achieve Turing universality. But, for conventional artificial neural networks, it was shown that 886 sigmoid function based processors are needed to achieve Turing universality^38^.

In the nervous system, synaptic plasticity forms the cell assemblies with the self-organization of neurons, which induces ordered or even synchronized neural dynamics replicating basic processes of long-term memory^39^,^40^. The self-organizing principle in the developing nervous system and its importance for preserving and continuing neural system development provide us insights on how neural-like networks might be reorganized and configured in response to environment changes. Enlightened by the biological fact, self-organizing artificial neural networks with unsupervised and supervised learning have been proposed and gain their popularity for visualisation and classification^41^,^42^. It is still an open problem as formulated by Gh Păun in ref. 43, to construct SN P systems with self-organization and to use the system to perform possible computer vision and pattern recognition tasks.

## Results

In this research, a novel variant of SN P systems, namely SN P systems with self-organization, is proposed and developed. The system initially has no synapse, but the synapses can be dynamically formed during the computation, which exhibits the self-organization behaviour. In the system, creation and deletion rules are used to create and delete synapses. The applications of synapse creation and deletion rules are controlled by the states of the involved neurons, i.e., the number of spikes contained in the neurons. The computational power of the system is investigated as well. As a result, it demonstrates that SN P systems with self-organization can compute and accept any set of Turing computable natural numbers. Moreover, with 87 neurons, the system can compute any Turing computable recursive function, ergo achieves Turing universality.

Before stating the results in mathematical forms, some notations should be introduced. *N*_*m*_*SPSO*_*all*_(*cre*_*h*_, *del*_*g*_, *rule*_*r*_) (resp. *N*_*m*_*SPSO*_*acc*_(*cre*_*h*′_, *del*_*g*′_, *rule*_*r*′_)) denotes the family of sets of numbers computed (resp. accepted) by SN P systems with self-organization of degree *m*, where *h* (resp. *h*′) indicates the maximal number of synapses that can be created using a synapse creation rule, *g* (resp. *g*′) is the maximal number of synapses that can be deleted by using a synapse deletion rule, *r* (resp. *r*′) is the maximal number of rules in each neuron, and the subscript *all* indicates the computation result is encoded by the number of spikes emitted into the environment (resp. the subscript *acc* indicates the system works in the accepting mode). If the parameters are not bounded, i.e., there is no limit imposed on them, then they are replaced with *. *NRE* denotes the family of Turing computable sets of numbers^44^.

The main results of this work can be mathematically depicted by the following theorems.

**Theorem 1.** N_*_SPSO_*all*_(cre_*_, del_*_, *rule*_5_) = *NRE.*

**Theorem 2.** N_*_SPSO_*acc*_(cre_*_, del_*_, *rule*_5_) = *NRE.*

**Theorem 3.** *There is a universal SN P system with self-organization having 87 neurons for computing functions.*

These results show that SN P systems with self-organization are powerful computing models, i.e., they are capable of doing what Turing machine can do. Also, they provide potential and theoretical feasibility of using SN P systems to solve real-life problems, such as pattern recognition and classification.

In SN P system with self-organization, it has no initially designed synapses. The synapses can be created or deleted according to the information contained in involved neurons during the computation. In previous work, it was found that the information diversing ability of synapses had some programable feature for SN P systems, but the computation power of SN P systems without initial synapses is an open problem. Although this is not the first time the feature of creating or deleting synapses investigated in SN P systems, see e.g. SN P systems with structural plasticity, it is quite the first attempt to construct SN P systems has no initial synapses.

## Methods

In this section, it starts by the mathematical definition of SN P system with self-organization, and then the computation power of SN P systems with self-organization is investigated as number generator, acceptor and function computing devices. It is proved in constructive ways that SN P systems with self-organization can compute and accept the family of sets of Turing computable natural numbers. With 87 neurons, such system can compute any Turing computable recursive function.

### Spiking Neural P Systems with Self-Organization

Before introducing the definition of SN P system with self-organization, some prerequisites of basic concepts of formal language theory^45^ are recalled.

For an alphabet *V*, V* denotes the set of all finite strings of symbols from *V*, the empty string is denoted by *λ*, and the set of all nonempty strings over *V* is denoted by *V*^+^. When *V* = {*a*} is a singleton, then we write simply a* and *a*^+^ instead of {*a*}*, {*a*}^+^. A regular expression over an alphabet *V* is defined as follows: (1) *λ* and each *a* ∈ *V* is a regular expression; (2) if *E*_1_ and *E*_2_ are regular expressions over *V*, then (*E*_1_)(*E*_2_), (*E*_1_) ∪ (*E*_2_), and (*E*_1_)^+^ are regular expressions over *V*; (3) nothing else is a regular expression over *V*.

For each regular expression *E*, a language *L*(*E*) is associated, defined in the following way: (1) *L*(*λ*) = {*λ*} and *L*(*a*) = {*a*}, for all *a* ∈ *V*, (2) *L*((*E*_1_)∪(*E*_2_)) = *L*(*E*_1_) ∪ *L*(*E*_2_), *L*((*E*_1_)(*E*_2_)) = *L*(*E*_1_)*L*(*E*_2_) and *L*((*E*_1_)^+^) = (*L*(*E*_1_))^+^ for all regular expressions *E*_1_, *E*_2_ over *V*. Unnecessary parentheses can be omitted when writing a regular expression, and (*E*)^+^ ∪ {*λ*} can also be written as E*. By *NRE* we denote the family of Turing computable sets of numbers. (*NRE* is the family of length sets of recursively enumerable languages–those recognized by Turing machines).

An SN P system with self-organization of degree *m *≥ 1 is a construct of the form


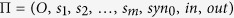


where

- *O* = {*a*} is a singleton, where *a* is called the spike;
- *σ*_1_, *σ*_2_, …, *σ*_*m*_ are neurons of the form *σ*_*i*_ = (*n*_*i*_, *R*_*i*_) with 1 ≤ *i* ≤ *m*, where

– 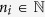 is the initial number of spikes contained in neuron *σ*_*i*_;

– *R*_*i*_ is a finite set of rules in neuron *σ*_*i*_ of the following three forms:

1. spiking rule: *E*/*a*^*c*^ → *a*^*p*^; *d*, where *E* is a regular expression over *O*, *d *≥ 0 and *c *≥ *p* ≥ 0;
2. synapse creation rule: *E*′/*a*^*c*′^ → +(*a*^*p*′^, *cre*(*i*)), where *E*′ is a regular expression over *O*, *cre*(*i*) ⊆ {*σ*_1_, *σ*_2_, …, *σ*_*m*_}/{*σ*_*i*_} and *c*′ ≥ *p*′ > 1;
3. synapse deletion rule: *E*″/*a*^*c*″^ → −(*λ*, *del*(*i*)), where *E*″ is a regular expression over *O*, *del*(*i*) ⊆ {*σ*_1_, *σ*_2_, …, *σ*_*m*_}/{*σ*_*i*_} and *c*″ ≥ 1;

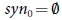
 is the initial set of synapses, which means no synapse is initially set; at any moment *t*, the set of synapses is denoted by *syn*_*t*_ ⊆ {1, 2, …, *m*} × {1, 2, …, *m*}.
- *in*, *out* ∈ {1, 2, …, *m*} indicates the input and output neuron, respectively.

A spiking rule of the form *E*/*a*^*c*^ → *a*^*p*^; *d* is applied as follows. If neuron *σ*_*i*_ contains *k* spikes, and *a*^*k*^ ∈ *L*(*E*), *k* ≥ *c*, then rule *E*/*a*^*c*^ → *a*^*p*^; *d* ∈ *R*_*i*_ can be applied. It means that *c* spikes are consumed and removed from neuron *σ*_*i*_, i.e., *k* − *c* spikes are remained, while the neuron emits *p* spikes to its neighboring neurons after *d* steps. (It is a common practice in membrane computing to have a global clock defined. The clock is used to mark the time of the whole system and ensure the system synchronization.) If *d* = 0, then the *p* spikes are emitted out immediately, if *d* = 1, then the *p* spikes are emitted in the next step, etc. If the rule is used in step *t* and *d* ≥ 1, then in steps *t*, *t* + 1, ..., *t* + *d* − 1 the neuron is closed (this corresponds to the refractory period from neurobiology), so that it cannot receive new spikes (if a neuron tries to send spikes to a neuron in close status, then these particular spikes will be lost). In the step *t* + *d*, the neuron fires and regains open status, so it can receive spikes (which can be used starting with the step *t* + *d* + 1, when the neuron can again apply rules). It is possible that *p* is associated with value 0. In this case, neuron *σ*_*i*_ consumes *c* spikes without emitting any spike. Spiking rule with *p* = 0 is also called forgetting rule, by which a pre-defined number of spikes can be removed out of the neuron. If *E* = *a*^*c*^, then the rule can be written in the simplified form *a*^*c*^ → *a*^*p*^; *d*, and if *d* = 0, then the rule can be simply written as *E*/*a*^*c*^ → *a*^*p*^.

Synapse creation and deletion rules are used to create and delete synapses during the computation. Synapse creation rule *E*′/*a*^*c*′^ → +(*a*^*p*′^, *cre*(*i*)) is applied as follows. If neuron *σ*_*i*_ has *k*′ spikes such that *a*^*k*′^ ∈ *L*(*E*′), *k*′ ≥ *c*′, then the synapse creation rule is applied with consuming *c*′ spikes, creating synapses to connect neuron *σ*_*i*_ to each neuron in *cre*(*i*) and emitting *p*′ spikes to each neuron in *cre*(*i*). If neuron *σ*_*i*_ has *k*″ spikes such that *a*^*k*″^ ∈ *L*(*E*″) and *k*″ ≥ *c*″, then synapse deletion rule *E*″/*a*^*c*″^ → −(*λ*, *del*(*i*)) is applied, removing *c*″ spikes from neuron *σ*_*i*_ and deleting all the synapses connecting neuron *σ*_*i*_ to the neurons from *del*(*i*). With the synapse creation and deletion rules, *E*′ and *E*″ are regular expressions over *O* = {*a*}, which regulate the application of synapse creation and deletion rules. This means that synapse creation and deletion rules can be used if and only if the neuron contain some particular numbers of spikes, i.e., the neuron is in some specific states. With the applications of synapse creation and deletion rules the system can dynamically rebuild its topological structure during the computation, which is herein defined as self-organization.

One neuron is specified as the input neuron, through which the system can read spikes from the environment. The output neuron has a synapse creation rule of the form *E*′/*a*^*c*′^ → +(*a*^*p*′^, {0}), where the environment is labelled by 0. By using the rule, the output neuron creates a synapse pointing to the environment, and then it can emit spikes into the environment along the created synapse.

For each time step, as long as there is one available rule in *R*_*i*_, neuron *σ*_*i*_ must apply the rule. It is possible that there are more than one rule that can be used in a neuron at some moment, since spiking rules, synapse creation rules and synapse deletion rules may be associated with regular languages (according to their regular expressions). In this case, the neuron will non-deterministically uses one of the enabled rules. The system works sequentially in each neuron (at most one rule from each *R*_*i*_ can be used), and if parallelism is designed for the system, all the neurons at the same system level have at least one enabled rule activated.

The configuration of the system at certain moment is defined by three major factors which are the number of spikes contained in each neuron, the number of steps to wait until it becomes open and the current set of synapses. With the notion, the initial configuration of the system is 〈*n*_1_/0, *n*_2_/0, …, *n*_*m*_/0, 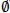. Using the spiking, forgetting, synapse creation and deletion rules as described above, we can define transitions among configurations. Any sequence of transitions starting from the initial configuration is called a computation. A computation halts, also called successful, if it reaches a configuration where no rule can be applied in any neuron in the system. For each successful computation of the system, a computation result is generated, which is total the number of spikes sent to the environment by the output neuron.

System Π generates a number *n* as follows. The computation of the system starts from the initial configuration and finally halts, emitting totally *n* spikes to the environment. The set of all numbers computed in this way by Π is denoted by *N*_*all*_(Π) (the subscript *all* indicates that the computation result is the total number of spikes emitted into the environment by the system). System Π can also work in the accepting mode. A number *n* is read through input neurons from the environment in form of spike train 10^*n*−1^1, which will be stored in a specified neuron *σ*_1_ in the form of *f*(*n*) spikes. If the computation eventually halts, then number *n* is said to be accepted by Π. The set of numbers accepted by Π is denoted by *N*_*acc*_(Π).

It is denoted by *N*_*m*_*SPSO*_*all*_(*cre*_*h*_, *del*_*g*_, *rule*_*r*_) (resp. *N*_*m*_*SPSO*_*acc*_(*cre*_*h*′_, *del*_*g*′_, *rule*_*r*′_)) the family of sets of numbers computed (resp. accepted) by SN P systems with self-organization of degree *m*, where *h* (resp. *h*′) indicates the maximal number of synapses that can be created with using a synapse creation rule, *g* (resp. *g*′) is the maximal number of synapses that can be deleted with using a synapse deletion rule, *r* (resp. *r*′) is the maximal number of rules in each neuron, and the subscript *all* indicates the computation result is encoded by the number of spikes emitted into the environment (resp. the subscript *acc* indicates the system works in a accepting mode). If the parameters are not bounded, i.e., there is no limit imposed on them, then they are replaced with *.

In order to compute a function *f* : N^*k*^ → **N** by SN P systems with self-organization, *k* natural numbers *n*_1_, *n*_2_, …, *n*_*k*_ are introduced in the system by reading from the environment a spike train (which is a binary sequence) 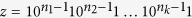. The input neuron has a synapse pointing from the environment, by which the spikes can enter it. The input neuron reads a spike in each step corresponding to a digit 1 from the string *z*; otherwise, no spike is received. Note that exactly *k* + 1 spikes are introduced into the system through the input neuron, i.e., after the last spike, it is assumed that no further spike is coming to the input neuron. The output neuron has a synapse pointing to the environment from it, by which the spikes can be emitted to the environment. The result of the computation is the total number of spikes emitted into the environment by the output neuron, hence producing *r* spikes with *r* = *f*(*n*_1_, *n*_2_, …, *n*_*k*_).

SN P systems with self-organization can be represented graphically, which is easier to understand than that in a symbolic way. A rounded rectangle with the initial number of spikes and rules is used to represent a neuron and a directed edge connecting two neurons represents a synapse.

In the following proofs, the notion of register machine is used. A register machine is a construct *M* = (*m*, *H*, *l*_0_, *l*_*h*_, *I*), where *m* is the number of registers, *H* is the set of instruction labels, *l*_0_ is the start label, *l*_*h*_ is the halt label (assigned to instruction *HALT*), and *I* is the set of instructions; each label from *H* labels only one instruction from *I*, thus precisele following forms:

- *l*_*i*_: (ADD(*r*), *l*_*j*_, *l*_*k*_) (add 1 to register *r* and then go to one of the instructions with labels *l*_*j*_, *l*_*k*_),
- *l*_*i*_: (SUB(*r*), *l*_*j*_, *l*_*k*_) (if register *r* is non-zero, then subtract 1 from it, and go to the instruction with label *l*_*j*_; otherwise, go to the instruction with label *l*_*k*_),
- *l*_*h*_: HALT (the halt instruction).

### As number generator

A register machine *M* computes a number *n* as follows. It starts by using initial instruction *l*_0_ with all registers storing number 0. When it reaches halt instruction *l*_*h*_, the number stored in register 1 is called the number generated or computed by register machine *M*. The set of numbers generated or computed by register machine *M* is denoted by *N*(*M*). It is known that register machines compute all sets of numbers which are Turing computable, hence they characterize *NRE*, i.e., *N*(*M*) = *NRE*, where *NRE* is the family of Turing computable sets of numbers^44^.

Without loss of generality, it can be assumed that in the halting configuration, all registers different from the first one are empty, and that the first register is never decremented during the computation (i.e., its content is only added to). When the power of two number generating devices *D*_1_ and *D*_2_ are compared, number zero is ignored; that is, *N*(*D*_1_) = *N*(*D*_2_) if and only if *N*(*D*_1_) − {0} = *N*(*D*_2_) − {0} (this corresponds to the usual practice of ignoring the empty string in language and automata theory).

**Theorem 4.** N_*_SPSO_*all*_(cre_*_, del_*_, *rule*_5_) = *NRE.*

#### Proof

It only has to prove *NRE*⊆N_*_SPSO_*all*_ (cre_*_, del_*_, *rule*_5_), since the converse inclusion is straightforward from the Turing-Church thesis (or it can be proved by the similar technical details in Section 8.1 in ref. 46, but is cumbersome). To achieve this, we use the characterization of *NRE* by means of register machines in the generative mode. Let us consider a register machine *M* = (*m*, *H*, *l*_0_, *l*_*h*_, *I*) defined above. It is assumed that register 1 of *M* is the output register, which is never decremented during the computation. For each register *r* of *M*, let *s*_*r*_ be the number of instructions of the form *l*_*i*_: (SUB(*r*), *l*_*j*_, *l*_*k*_), i.e., the number of SUB instructions acting on register *r*. If there is no such SUB instruction, then *s*_*r*_ = 0, which is the case for the first register *r* = 1. In what follows, a specific SN P system with self-organization Π is constructed to simulate register machine *M*.

System Π consists of three modules–ADD, SUB and FIN modules. The ADD and SUB modules are used to simulate the operations of ADD and SUB instructions of *M*; and the FIN module is used to output a computation result.

In general, with any register *r* of *M*, a neuron *σ*_*r*_ in system Π is associated; the number stored in register *r* is encoded by the number of spikes in neuron *σ*_*r*_. Specifically, if register *r* stores number *n* ≥ 0, then there are 5*n* spikes in neuron *σ*_*r*_. For each label *l*_*i*_ of an instruction in *M*, a neuron 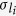 is associated. During the simulation, when neuron 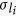 receives 6 spikes, it becomes active and starts to simulate instruction *l*_*i*_: (OP(*r*), *l*_*j*_, *l*_*k*_) of *M*: the process starts with neuron 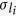 activated, operates on the number of spikes in neuron *σ*_*r*_ as requested by OP, then sends 6 spikes into neuron 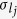 or 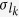, which becomes active in this way. Since there is no initial synapse in system Π, some synapses are created to pass spikes to target neurons with synapse creation rules, after that the created synapses will be deleted when simulation completes by synapse deletion rules. When neuron 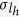 (associated with the halting instruction *l*_*h*_ of *M*) is activated, a computation in *M* is completely simulated by system Π.

The following describes the works of ADD, SUB, and FIN modules of the SN P systems with self-organization.

**Figure 1:**
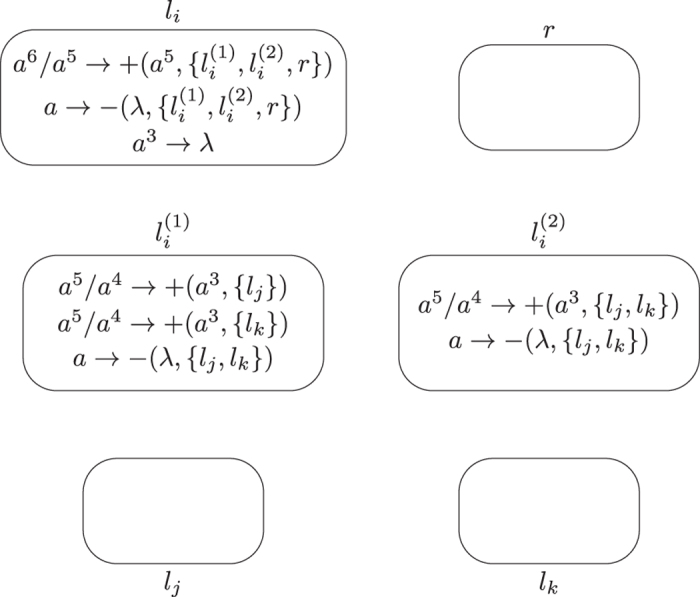
Module ADD simulating the ADD instruction.

**Module ADD (shown in** Fig. 1**): Simulating the ADD instruction** *l*_*i*_: (ADD(*r*), *l*_*j*_, *l*_*k*_).

Initially, there is no synapse in system Π, and all the neurons have no spike with exception that neuron 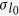 has 6 spikes. This means system Π starts by simulating initial instruction *l*_0_. Let us assume that at step *t*, an instruction *l*_*i*_: (ADD(*r*), *l*_*j*_, *l*_*k*_) has to be simulated, with 6 spikes present in neuron 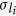 (like 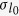 in the initial configuration) and no spike in any other neurons, except in those neurons associated with registers.

At step *t*, neuron 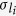 has 6 spikes, and synapse creation rule 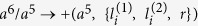 is applied in 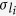, it generates three synapses connecting neuron 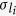 to neurons 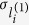, 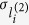 and *σ*_*r*_. Meanwhile, it consumes 5 spikes (one spike remaining) and sends 5 spikes to each of neurons 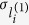, 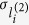 and *σ*_*r*_. The number of spikes in neuron *σ*_*r*_ is increased by 5, which simulates adding 1 to register *r* of *M*. At step *t* + 1, neuron 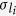 deletes the three synapses created at step *t* by using rule 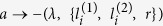. At the same moment, neuron 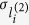 uses synapse creation rule *a*^5^/*a*^4^ → + (*a*^3^, {*l*_*j*_, *l*_*k*_}), and creates two synapses to neurons 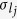 and 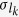, as well as sends 3 spikes to each of the two neurons. At step *t* + 2, neuron 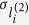 deletes the two synapses by using synapse deletion rule *a* → − (*λ*, {*l*_*j*_, *l*_*k*_}). In neuron 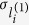, there are 5 spikes at step *t* + 1 such that both of synapse creation rules *a*^5^/*a*^4^ → + (*a*^3^, {*l*_*j*_}) and *a*^5^/*a*^4^ → + (*a*^3^, {*l*_*k*_}) are enabled, but only one of them is non-deterministically used.

– If rule *a*^5^/*a*^4^ → + (*a*^3^, {*l*_*j*_}) is chosen to use, neuron 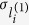 creates a synapse and sends 3 spikes to neuron 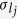. In this case, neuron 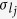 accumulates 6 spikes, which means system Π starts to simulate instruction *l*_*j*_ of *M*. One step later, with one spike inside neuron 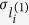 uses rule *a* → − (*λ*, {*l*_*j*_, *l*_*k*_}) to delete the synapse to neuron 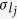, and neuron 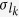 removes the 3 spikes (from neuron 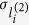) by the forgetting rule *a*^3^ → *λ*.

– If rule *a*^5^/*a*^4^ → + (*a*^3^, {*l*_*k*_}) is selected to apply, neuron 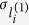 creates a synapse and sends 3 spikes to neuron 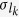. Neuron 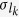 accumulates 6 spikes, which indicates system Π goes to simulate instruction *l*_*k*_ of *M*. One step later, neuron 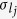 removes the 3 spikes by using forgetting rule *a*^3^ → *λ*, and the synapse from neuron 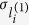 to 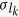 is deleted by using rule *a* → − (*λ*, {*l*_*j*_, *l*_*k*_}) in neuron 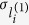.

**Figure 2:**
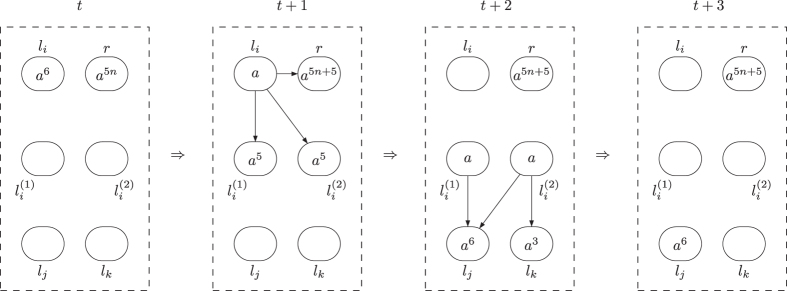
The dynamic transformation of topological structure and the numbers of spikes in neurons of ADD module during the ADD instruction simulation with neuron 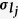 finally activated.

**Figure 3:**
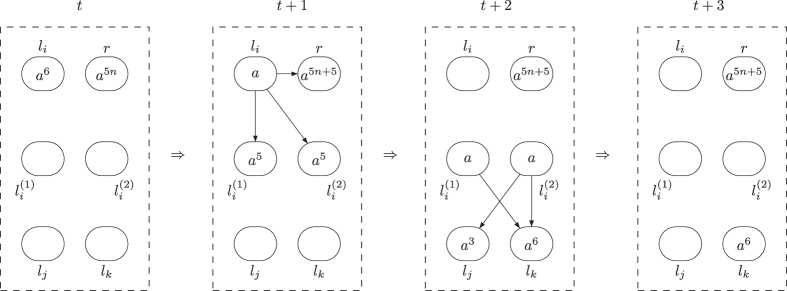
The dynamic transformation of topological structure and the numbers of spikes in neurons of ADD module during the ADD instruction simulation with neuron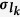 finally activated.

Therefore, from firing neuron 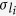, system Π adds 5 spikes to neuron *σ*_*r*_ and non-deterministically activates one of the neurons 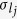 and 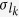, which correctly simulates the ADD instruction *l*_*i*_: (ADD(*r*), *l*_*j*_, *l*_*k*_). When the simulation of ADD instruction is completed, the ADD module returns to its initial topological structure, i.e., there is no synapse in the module. The dynamic transformation of topological structure and the numbers of spikes in neurons of ADD module during the ADD instruction simulation with neuron 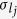 or 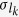 finally activated is shown in Figs 2 and 3. In the figures, the spiking rules are omitted for clear illustration, neurons are represented by circles with the number of spikes and directed edges is used to represent the synapses.

**Figure 4:**
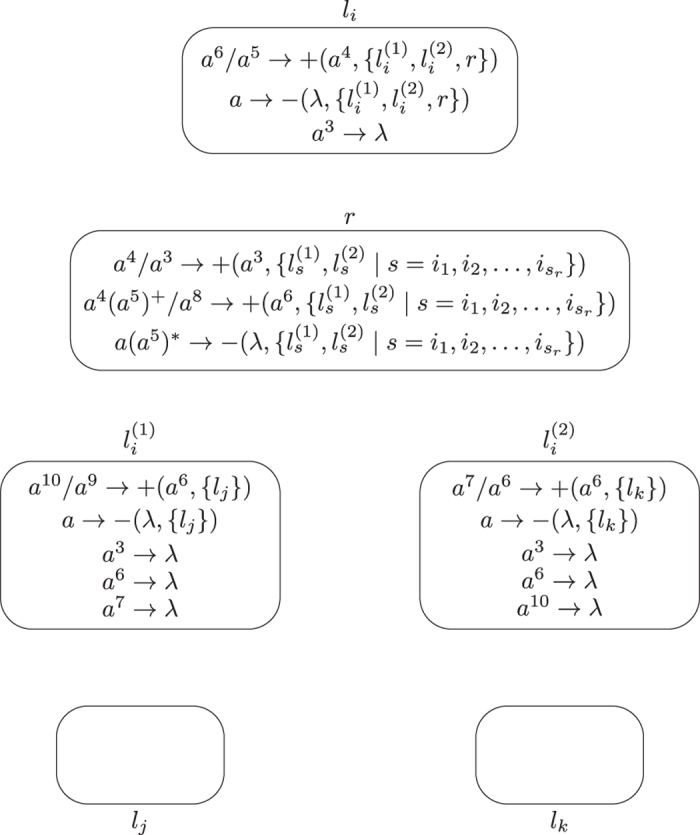
Module SUB simulating the SUB instruction.

**Module SUB (shown in** Fig. 4**): Simulating the SUB instruction** *l*_*i*_: (SUB(*r*), *l*_*j*_, *l*_*k*_).

Given starting time stamp *t*, system Π simulates a SUB instruction *l*_*i*_: (SUB(*r*), *l*_*j*_, *l*_*k*_). Let *s*_*r*_ be the number of SUB instructions acting on register *r* and the set of labels of instructions acting on register *r* be 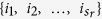. Obviously, it holds 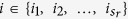.

At step *t*, neuron 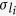 has 6 spikes, and becomes active by using synapse creation rule 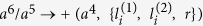, creating synapses and sending 4 spikes to each of neurons 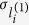, 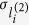 and *σ*_*r*_. With 4 spikes inside, neurons 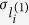 and 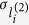 keep inactive at step *t* + 1 because no rule can be used. In neuron *σ*_*r*_, it has the following two cases.

– If neuron *σ*_*r*_ has 5*n* (*n* > 0) spikes (corresponding to the fact that the number stored in register *r* is *n*, and *n* > 0), then by receiving 4 spikes from neuron 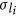, it accumulates 5*n* + 4 spikes and becomes active by using rule 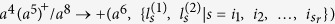 at step *t* + 1. It creates a synapse to each of neurons 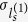 and 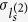 with 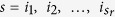 and sending 6 spikes to the neurons. By consuming 8 spikes, the number of spikes in neuron *σ*_*r*_ becomes 5*n* + 4 − 8 = 5(*n* − 1) + 1 (*n* ≥ 0) such that rule 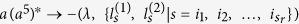 is enabled and applied at step *t* + 2. With application of the rule, neuron *σ*_*r*_ removes the synapses from neuron *σ*_*r*_ to neurons 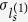 and 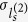, 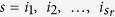. Meanwhile, neurons 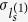 and 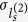 with *s* ≠ *i* remove the 6 spikes by using forgetting rule *a*^6^ → *λ*, and neuron 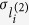 removes the 10 spikes by forgetting rule *a*^10^ → *λ*. Neuron 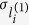 accumulates 10 spikes (4 spikes from neuron 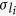 and 6 spikes from neuron *σ*_*r*_), and rule *a*^10^/*a*^9^ → + (*a*^6^, {*l*_*j*_}) is applied at step *t* + 2, creating a synapse and sending 6 spikes to neuron 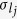. In this case, neuron 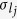 receives 6 spikes, which means system Π starts to simulate instruction *l*_*j*_ of *M*. One step later, the synapse from neuron 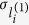 to neuron 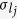 is deleted by using synapse deletion rule *a* → − (*λ*, {*l*_*j*_}).

– If neuron *σ*_*r*_ has no spike (corresponding to the fact that the number stored in register *r* is 0), then after receiving 4 spikes from neuron 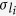, it has 4 spikes and rule 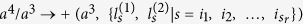 is used, creating a synapse to each of neurons 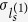 and 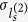 with 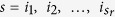 and sending 3 spikes to the neurons. Neuron *σ*_*r*_ remains one spike, and synapse deletion rule 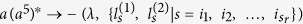 is applied at step *t* + 2, removing the synapses from neuron *σ*_*r*_ to neurons 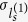 and 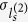, 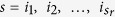. At the same moment, neurons 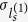 and 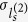 with *s* ≠ *i* remove the 3 spikes by using forgetting rule *a*^3^ → *λ*, and neuron 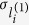 removes 7 spikes using spiking rule *a*^7^ → *λ*. Having 7 spikes, Neuron 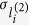 becomes active by using rule *a*^7^/*a*^6^ → + (*a*^6^, {*l*_*k*_}) at step *t* + 2, creating a synapse to neuron 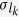 and sending 6 spikes to neuron 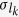. In this case, neuron 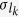 receives 6 spikes, which means system Π starts to simulate instruction *l*_*k*_ of *M*. At step *t* + 3, neuron 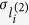 uses rule *a* → − (*λ*, {*l*_*k*_}) to remove the synapse to neuron 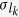.

The simulation of SUB instruction performs correctly: System Π starts from 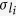 having 6 spikes and becoming active, and ends in neuron 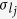 receiving 6 spikes (if the number stored in register *r* is great than 0 and decreased by one), or in neuron 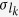 receiving 6 spikes (if the number stored in register *r* is 0).

**Figure 5:**
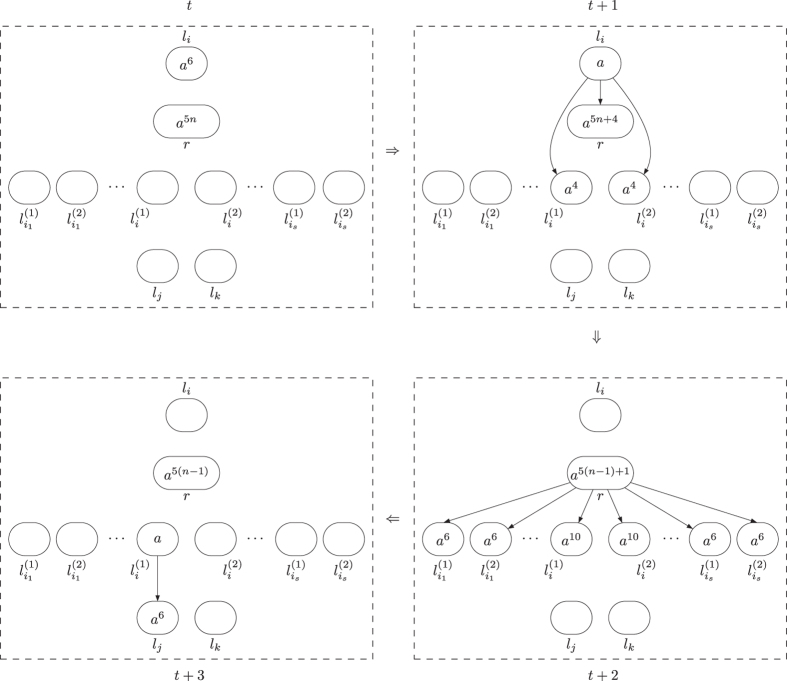
The dynamic transformation of topological structure and the numbers of spikes in involved neurons in the SUB instruction simulation with neuron 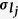 finally activated.

**Figure 6:**
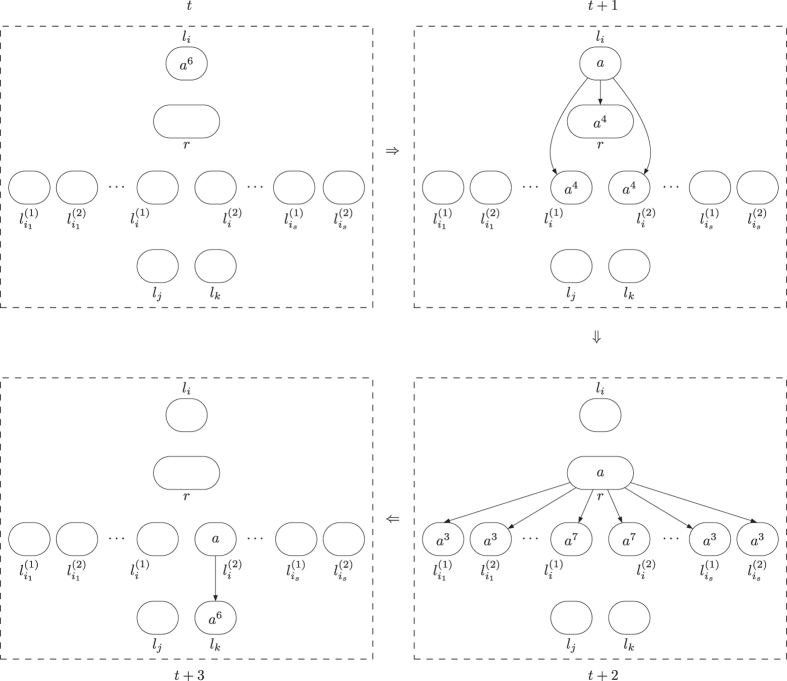
The dynamic transformation of topological structure and the numbers of spikes in involved neurons in the SUB instruction simulation with neuron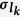 finally activated.

When the simulation of SUB instruction is completed, the SUB module returns to its initial topological structure, i.e., there is no synapse in the module. The dynamic transformation of topological structure and the numbers of spikes in involved neurons in the SUB instruction simulation with neuron 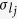 (resp. neuron 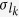) finally activated is shown in Fig. 5 (resp. Fig. 6).

**Figure 7:**
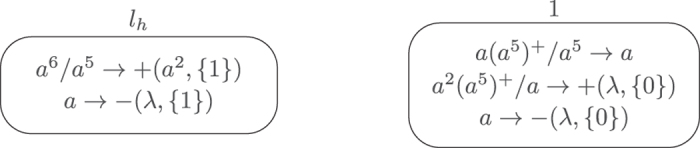
Outputting the computation result.

**Module FIN** (shown in Fig. 7) – outputting the result of computation.

Assume that at step *t* the computation in *M* halts, i.e., the halting instruction is reached. In this case, neuron 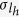 in Π receives 6 spikes. At that moment, neuron *σ*_1_ contains 5*n* spikes, for the number *n* ≥ 1 stored in register 1 of *M*. With 6 spikes inside, neuron 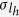 becomes active by using rule *a*^6^/*a*^5^ → +(*a*^2^, {1}), creating a synapse to neuron *σ*_1_ and sending 2 spikes to neuron *σ*_1_. Neuron 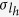 ends with one spike, and rule *a* → −(*λ*, {1}) is used, removing the synapse to neuron *σ*_1_ one step later.

After neuron *σ*_1_ receives the 2 spikes from neuron 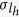, the number of spikes in neuron *σ*_1_ becomes 5*n* + 2 and rule *a*^2^(*a*^5^)^+^/*a* → +(*λ*, {0}) is enabled and applied at step *t* + 2. By using the rule, neuron *σ*_1_ consumes one spike and creates a synapse to the environment. Neuron *σ*_1_ contains 5*n* + 1 spikes such that spiking rule *a*(*a*^5^)^+^/*a*^5^ → *a* is used, consuming 5 spikes and emitting one spike to the environment at step *t* + 3. Note that the number of spikes in neuron *σ*_1_ becomes 5(*n* − 1) + 1. So, if the number of spikes in neuron *σ*_1_ is not one, then neuron *σ*_1_ will fire again in the next step sending one spike into the environment. In this way, neuron *σ*_1_ can fire for *n* times, i.e., until the number of spikes in neuron *σ*_1_ reaches one. For each time when neuron *σ*_1_ fires, it sends one spike into the environment. So, in total, neuron *σ*_1_ sends *n* spikes into the environment, which is exactly the number stored in register 1 of *M* at the moment when the computation of *M* halts. When neuron *σ*_1_ has one spike, rule *a* → −(*λ*, {0}) is used to remove the synapse from neuron *σ*_1_ to the environment, and system Π eventually halts.

**Figure 8:**
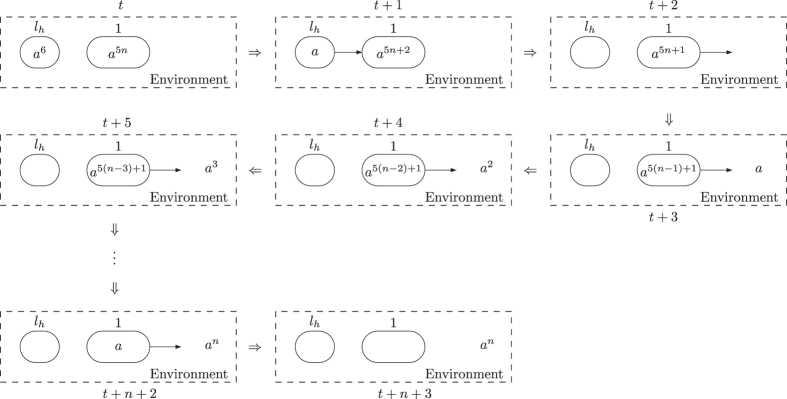
The dynamic transformation of topological structure of the FIN module and the numbers of spikes in the neurons of FIN module and the environment.

The dynamic transformation of topological structure of the FIN module and the numbers of spikes in the neurons of FIN module and in the environment are shown in Fig. 8.

Based on the description of the work of system Π above, the register machine *M* is correctly simulated by system Π, i.e., *N*(*M*) = *N*_*all*_(Π). We can check that each neuron in system Π has at most three rules, and no limit is imposed on the numbers of neurons and the synapses that can be created (or deleted) by using one synapse creation (or deletion) rule. Therefore, it concludes N_*_SPSO_*all*_(cre_*_, del_*_, *rule*_5_) = *NRE*.

This concludes the proof.

### As number acceptor

Register machine can work in the accepting mode. Number *n* is accepted by register machine *M*′ as follows. Initially, number *n* is stored in the first register of *M*′ and all the other registers are empty. If the computation starting in this configuration eventually halts, then the number *n* is said to be accepted by register machine *M*′. The set of numbers accepted by register machine *M*′ is denoted by *N*_*acc*_(*M*′). It is known that all the sets of numbers in *NRE* can be accepted by register machine *M*′, even using the deterministic register machine; i.e. the machine with the ADD instructions of the form *l*_*i*_: (ADD(*r*), *l*_*j*_, *l*_*k*_) where *l*_*j*_ = *l*_*k*_ (in this case, the instruction is written in the form *l*_*i*_: (ADD(*r*), *l*_*j*_))^44^.

**Theorem 5.** N_*_SPSO_*acc*_(cre_*_, del_*_, *rule*_5_) = *NRE.*

#### Proof

It only has to prove *NRE* ⊆ N_*_SPSO_*acc*_ (cre_*_, del_*_, *rule*_5_), since the converse inclusion is straightforward from the Turing-Church thesis. In what follows, an SN P system Π′ with self-organization working in accepting mode is constructed to simulate a deterministic register machine *M*′ = (*m*, *H*, *l*_0_, *l*_*h*_, *I*) working in the acceptive mode. Actually, the proof is given by modifying the proof of Theorem 4.

Each register *r* of *M*′ is associated with a neuron *σ*_*r*_ in system Π′, and for each instruction *l*_*i*_ of *M*′ a neuron 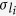 is associated. A number *n* stored in register *r* is represented by 5*n* spikes in neuron *σ*_*r*_.

**Figure 9:**
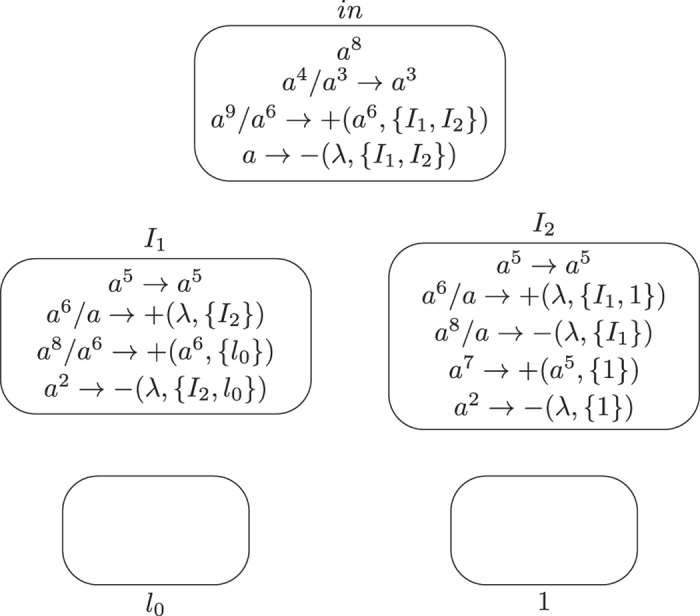
The INPUT module of system Π′.

The system Π′ consists of an INPUT module, deterministic ADD and SUB modules. The INPUT module is shown in Fig. 9, where all neurons are initially empty with the exception that input neuron *σ*_*in*_ has 8 spikes. Spike train 10^*n*−1^1 is introduced into the system through input neuron *σ*_*in*_, where the internal between the two spikes in the spike train is (*n* + 1) − 1 = *n*, which indicates that number *n* is going to be accepted by system Π′.

Assuming at step *t* neuron *σ*_*in*_ receives the first spike. At step *t* + 1, neuron *σ*_*in*_ contains 9 spikes, and rule *a*^9^/*a*^6^ → +(*a*^6^, {*I*_1_, *I*_2_}) is used, creating a synapse from neuron *σ*_*in*_ to neurons 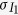 and 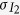. Meanwhile, neuron *σ*_*in*_ sends 6 spikes to the two neurons. In neuron *σ*_*in*_, 6 spikes are consumed and 3 spikes remain. With 6 spikes inside, neurons 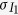 and 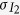 become active at step *t* + 2. Neuron 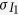 uses rule *a*^6^/*a* → +(*λ*, {*I*_2_}) to create a synapse to neuron 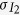; and neuron 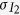 uses rule *a*^6^/*a* → +(*λ*, {*I*_1_, 1}) to create a synapse to each of neurons 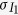 and *σ*_1_. Each of neurons 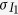 and 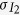 has 5 spikes left. From step *t* + 3 on, neurons 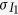 and 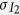 fire and begin to exchange 5 spikes between them. In this way, neuron *σ*_1_ receives 5 spikes from neurons 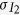 at each step.

At step *t* + *n*, neuron *σ*_*in*_ receives the second spike from the environment, accumulating 4 spikes inside. At step *t* + *n* + 1, neuron *σ*_*in*_ fires for the second time by using spiking rule *a*^4^/*a*^3^ → *a*^3^, sending 3 spikes to neurons 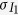 and 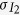. Each of neurons 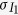 and 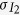 accumulates 8 spikes. At step *t* + *n* + 2, neuron 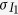 uses synapse creation rule *a*^8^/*a*^6^ → +(*a*^6^, {*l*_0_}), creating a synapse to neuron 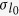 and sending 6 spikes to neuron 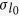. This means that system Π′ starts to simulate the initial instruction *l*_0_ of register machine *M*′. Meanwhile, neuron 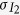 uses synapse deletion rule *a*^8^/*a* → −(*λ*, {*I*_1_}), removing the synapse from neuron .. to neuron 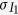. In the next step, neuron 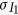 creates a synapse to neuron *σ*_1_ and sends 5 spikes to neuron *σ*_1_ by using rule *a*^7^ → +(*a*^5^, {1}).

From step *t* + 3 to *t* + *n* + 1, neuron *σ*_1_ receives 5 spikes in each step from neuron 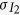, thus in total accumulating 5(*n* − 1) spikes. Neuron *σ*_1_ receives no spike at step *t* + *n* + 2, and gets 5 spikes from neuron 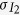 at step *t* + *n* + 3. After that, no more spikes are sent to neuron 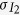. Neuron *σ*_1_ contains 5*n* spikes, which indicates the number to be accepted by register machine *M*′ is *n*. At step *t* + *n* + 4, neuron 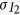 uses rule *a*^2^ → −(*λ*, {1}), deleting the synapse to neuron *σ*_1_.

**Figure 10:**
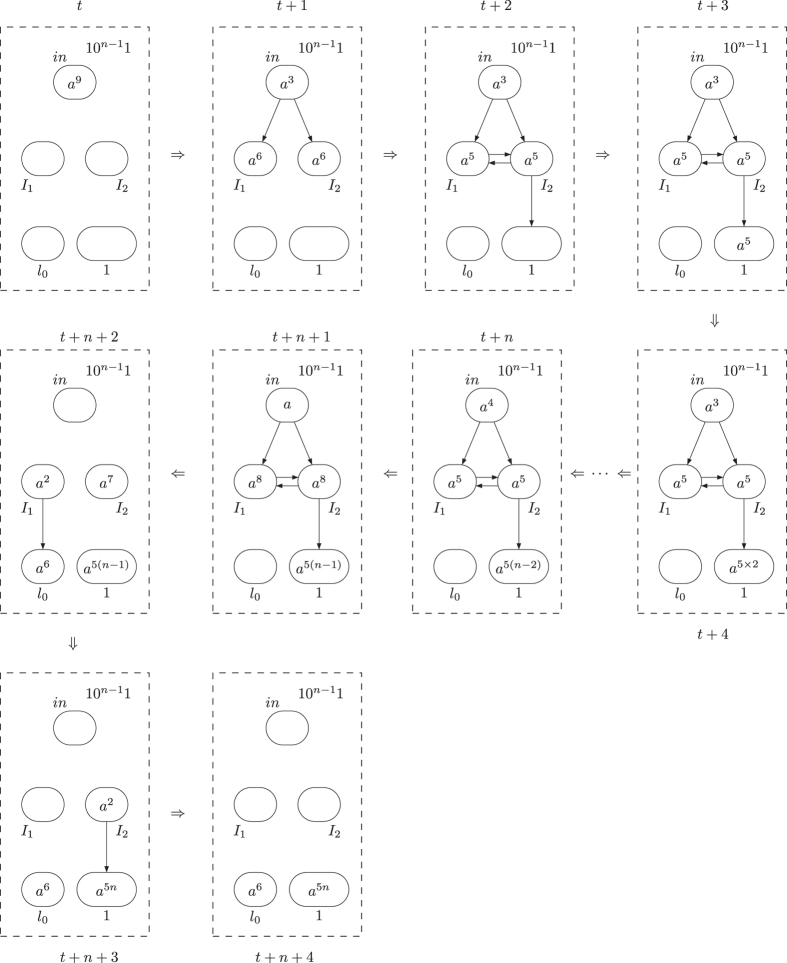
The dynamic transformation of topological structure of INPUT module and the numbers of spikes in the neurons of INPUT module.

The dynamic transformation of topological structure of INPUT module and the numbers of spikes in the neurons of INPUT module are shown in Fig. 10.

**Figure 11:**
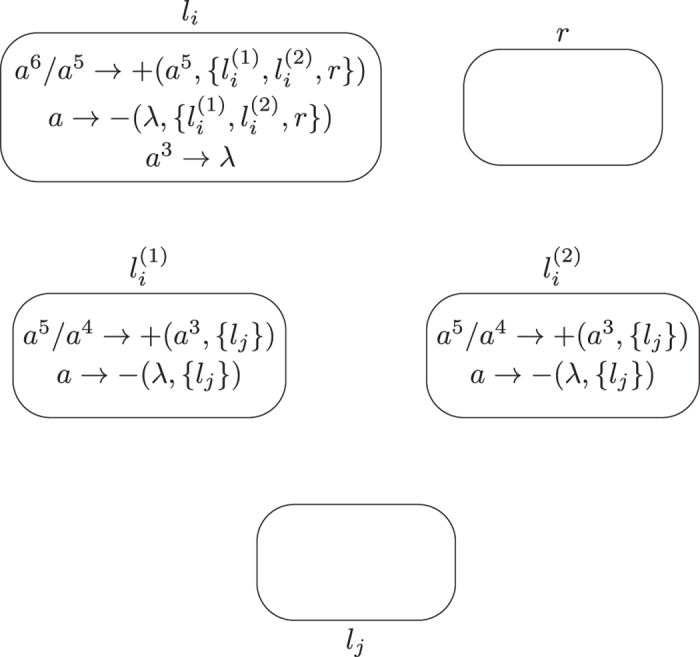
The deterministic ADD module of system Π′.

The deterministic ADD module is shown in Fig. 11, whose function is rather clear. By receiving 6 spikes, neuron 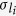 becomes active, creating a synapse and sending 5 spikes to each of neurons *σ*_*r*_, 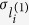 and 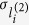. The number of spikes in neuron *σ*_*r*_ is increased by 5, which simulates the number stored in register 1 is increased by one. In the next step, neuron 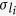 uses rule 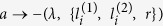, removing the synapses from neuron 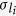 to neurons *σ*_*r*_, 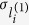 and 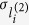. In neurons 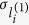 and 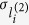, there are 5 spikes. The two neurons become active by using rule *a*^5^/*a*^4^ → +(*a*^3^, {*l*_*j*_}). Each of them creates a synapse to neuron 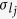 and emits 3 spikes to neuron 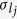. In this way, neuron 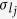 accumulates 6 spikes inside, which means the system Π′ goes to simulate instruction *l*_*j*_ of *M*′. The synapses from neuron 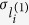 and 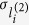 to neuron 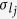 will be removed by using synapse deletion rule *a* → −(*λ*, {*l*_*j*_}) in neurons 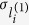 and 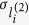.

Module SUB remains unchanged, as shown in Fig. 4. Module FIN is removed, with neuron 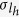 remaining in the system, but having no rule inside. When neuron 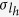 receives 6 spikes, it means that the computation of register machine *M*′ reaches instruction *l*_*h*_ and stops. Having 6 spikes inside, neuron 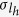 cannot become active for no rule can be used. In this way, the work of system Π′ halts.

Based on the description of the implementation of system Π′ above, it is clear that the register machine *M*′ in acceptive mode is correctly simulated by the system Π′ working in acceptive mode, i.e., *N*_*acc*_(*M*′) = *N*_*acc*_(Π′).

We can check that each neuron in system Π′ has at most five rules, and no limit is imposed on the numbers of neurons and the synapses that can be created (or deleted) with using one synapse creation (or deletion) rule. Therefore, it concludes N_*_SPSO_*acc*_(cre_*_, del_*_, *rule*_5_) = *NRE*.

### As function computing device

A register machine *M* can compute a function *f* : N^*k*^ → N as follows: the arguments are introduced in special registers *r*_1_, *r*_2_, …, *r*_*k*_ (without loss of the generality, it is assumed that the first *k* registers are used). The computation starts with the initial instruction *l*_0_. if the register machine halts, i.e., reaches HALT instruction *l*_*h*_, the value of the function is placed in another specified register, labelled by *r*_*t*_, with all registers different from *r*_*t*_ storing number 0. The partial function computed by a register machine *M* in this way is denoted by *M*(*n*_1_, *n*_2_, …, *n*_*k*_). All Turing computable functions can be computed by register machine in this way.

Several universal register machines for computing functions were defined. Let (*φ*_0_, *φ*_1_,…) be a fixed admissible enumeration of the unary partial recursive functions. A register machine *M*_*u*_ is said to be universal if there is a recursive function *g* such that for all natural numbers *x*, *y* we have *φ*_*x*_(*y*) = *M*_*u*_(*g*_(*x*)_, *y*). As addressed by Minsky, universal register machine can compute any *φ*_*x*_(*y*) by inputting a couple of numbers *g*(*x*) and *y* in registers 1 and 2, and the result can be obtained in register 0^47^.

**Figure 12:**
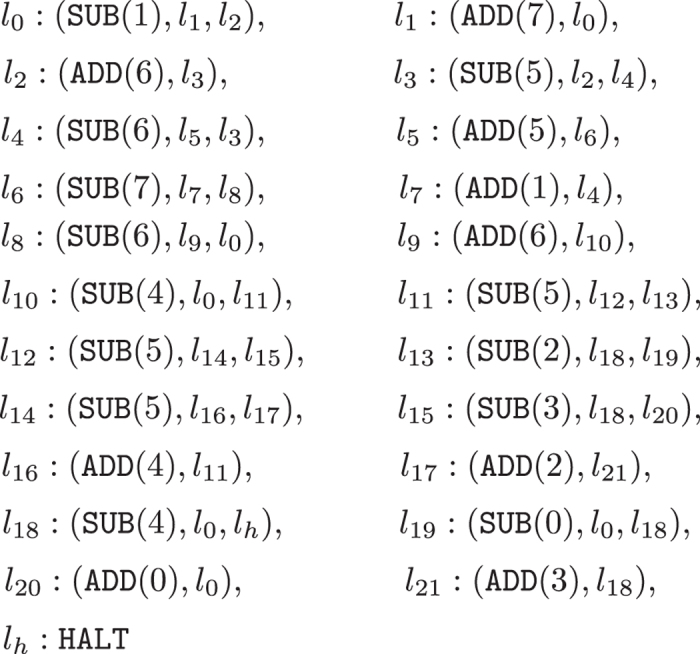
The universal register machine *M*_*u*_ from^47^.

In the following proof of universality, a specific universal register machine *M*_*u*_ from^47^ is used, the machine *M*_*u*_ = (8, *H*, *l*_0_, *l*_*h*_, *I*) presented in Fig. 12. In this universal register machine *M*_*u*_, there are 8 registers (numbered from 0 to 7) and 23 instructions, and the last instruction is the halting one. As described above, the input numbers (the “code” of the partial recursive function to compute and the argument for this function) are introduced in registers 1 and 2, and the result is outputted in register 0 when the machine *M*_*u*_ halts.

A modification is necessary to be made in *M*_*u*_, because the subtraction operation in the register where the result is placed is not allowed in the construction of the previous Theorems, but register 0 of *M*_*u*_ is subject of such operations. That is why an extra register is needed - labeled with 8 - and the halt instruction *l*_*h*_ of *M*_*u*_ should be replaced by the following instructions:


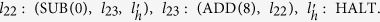


Therefore, the modified universal register machine 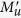 has 9 registers, 24 ADD and SUB instructions, and 25 labels. The result of a computation of 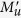 is stored in register 8

**Theorem 6** *There is a universal SN P system with self-organization having 87 neurons for computing functions.*

#### Proof

An SN P system with self-organization Π″ is constructed to simulate the computation of the universal register machine 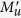. Specifically, the system Π″ consists of deterministic ADD modules, SUB modules, as well as an INPUT module and an OUTPUT module. The deterministic ADD module shown in Fig. 11 and SUB module shown in Fig. 4 can be used here to simulate the deterministic ADD instruction and SUB instruction of 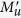. The INPUT module introduces the necessary spikes into the system by reading a spike train from the environment, and the OUTPUT module outputs the computation result.

With each register *r* of 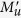, a neuron *σ*_*r*_ in system Π″ is associated; the number stored in register *r* is encoded by the number of spikes in neuron *σ*_*r*_. If register *r* holds the number *n* ≥ 0, then neuron *σ*_*r*_ contains 5*n* spikes. With each instruction *l*_*i*_ in .., a neuron 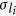 in system Π″ is associated. If neuron 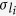 has 6 spikes inside, it becomes active and starts to simulate the instruction *l*_*i*_. When neuron 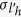 (associated with the label *l*′_*h*_ of the halting instruction of 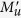) receives 6 spikes, the computation in 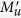 is completely simulated by the system Π″; the number of spikes emitted into the environment from the output neuron, i.e., neuron *σ*_8_, corresponds to the result computed by 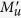 (stored in register 8).

**Figure 13:**
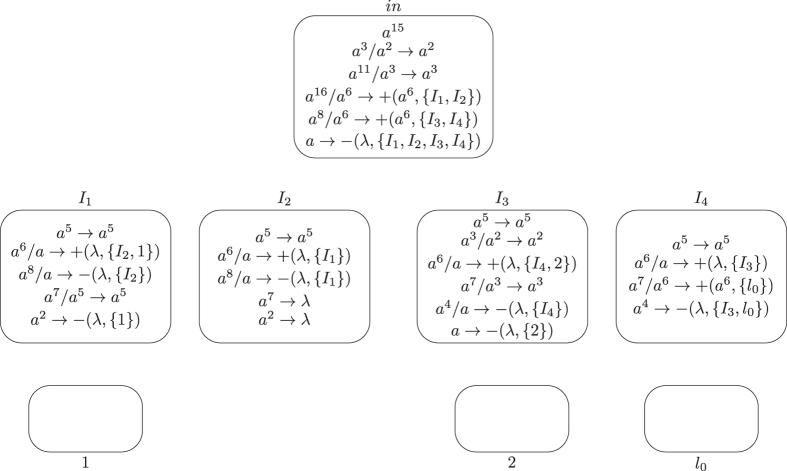
The INPUT module of system Π″.

The tasks of loading 5*g*(*x*) spikes in neuron *σ*_1_ and 5*y* spikes in neuron *σ*_2_ by reading the spike train 10^*g*(*x*)−1^10^*y*−1^1 through input neuron *σ*_*in*_ can be carried out by the INPUT module shown in Fig. 13.

Initially, all the neurons contain no spike inside, with the exception that neuron *σ*_*in*_ has 15 spikes. It is assumed at step *t* neuron *σ*_*in*_ reads the first spike from the environment. With 16 spikes inside, neuron *σ*_*in*_ becomes active by using rule *a*^16^/*a*^6^ → +(*a*^6^, {*I*_1_, *I*_2_}) at step *t* + 1. It creates a synapse and sends 6 spikes to each of neurons 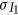 and 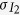. Subsequently, neuron *σ*_*in*_ keeps inactive (for no rule can be used) until the second spike arrives at step *t* + *g*(*x*). Neuron 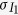 has 6 spikes and uses rule *a*^6^/*a* → +(*λ*, {*I*_2_, 1}) at step *t* + 2 it creates a synapse to neurons 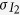 and *σ*_1_ and sends 5 spikes to each of the two neurons. Meanwhile, neuron 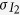 creates a synapse to neuron 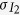 and sends 5 spikes to it. From step *t* + 3 on, neuron 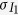 sends 5 spikes to neuron 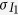 and exchanges 5 spikes with neuron 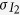 in each step.

At step *t* + *g*(*x*), neuron *σ*_*in*_ receives the second spike from the environment. By then it accumulates 11 spikes inside. At step *t* + *g*(*x*) + 1, neuron *σ*_*in*_ fires by using spiking rule *a*^11^/*a*^3^ → *a*^3^, and sends 3 spikes to neurons 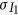 and 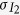. Each of neurons 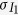 and .. contains 8 spike, which will be remained in *σ*_*in*_.

At step *t* + *g*(*x*) + 2, neuron 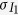 applies synapse deletion rule *a*^8^/*a* → −(*λ*, {*I*_1_}) and removes the synapse to neuron 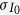, meanwhile neuron 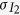 removes the synapse to neuron 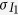. The two neurons stop to exchange spikes with each other. At step *t* + *g*(*x*) + 3, neuron 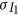 has 7 spikes and fires by using spiking rule *a*^7^/*a*^5^ → *a*^5^, and sends 5 spikes to neuron *σ*_1_. In the next step, neuron 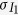 removes the synapse to neuron *σ*_1_, and cannot send spikes to neuron *σ*_1_.

In general, in each step from step *t* + 3 to *t* + *g*(*x*) +1, neuron *σ*_1_ receives 5 spikes from neuron 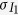, in total receiving 5(*g*(*x*) − 1) spikes; at step *t* + *g*(*x*) + 2, no spike arriving in neuron *σ*_1_, and at step *t* + *g*(*x*) + 3, 5 spikes reaching neuron *σ*_1_. In this way, neuron *σ*_1_ accumulates 5*g*(*x*) spikes, which simulates number *g*(*x*) is stored in register 1 of 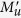.

At step *t* + *g*(*x*) + 2, neuron *σ*_*in*_ contains 8 spikes such that rule *a*^8^/*a*^6^ → + (*a*^6^, {*I*_3_, *I*_4_}) is used, creating a synapse and sends 6 spikes to each of neurons 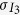 and 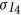. At step *t* + *g*(*x*) + 3, neurons 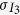 and 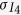 create synapses to each other, meanwhile neuron 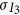 creates a synapse to neuron *σ*_2_. From step *t* + *g*(*x*) + 4 on, neuron 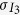 begins to exchange 5 spikes with 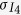 and send 5 spikes to neuron *σ*_2_ in each step. At step *t* + *g*(*x*) + *y*, neuron *σ*_*in*_ receives the third spike from the environment, accumulating 3 spikes inside. One step later, it fires by using spiking rule *a*^3^/*a*^2^ → *a*^2^, sending 2 spikes to neurons 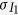, 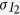, 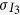 and 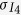. With 2 spikes inside, neuron *σ*_1_ removes its synapse to neuron *σ*_1_ by rule *a*^2^ → −(*λ*, {1}); while neuron 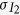 forgets the two spikes by forgetting rule *a*^2^ → *λ*. By receiving 2 spikes from neuron *σ*_*in*_, neurons 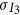 and 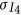 contain 7 spikes. At step *t* + *g*(*x*) + *y* + 2, neuron 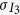 fires by using rule *a*^7^/*a*^3^ → *a*^3^ and sends 3 spikes to neurons *σ*_2_ and 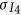. The number of spikes in neuron *σ*_2_ is 5(*y* − 1) + 3. Neuron 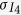 consumes 6 spikes, creates a synapse and sends 6 spikes to neuron 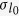. This means system Π'' starts to simulate the initial instruction *l*_0_ of 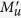.

At step *t* + *g*(*x*) + *y* + 3, neuron 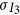 has 4 spikes, and it becomes active by rule *a*^4^/*a* → −(*λ*, {*I*_4_}), and removes its synapse to neuron 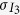 and ends with 3 spikes. In the next step, neuron 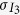 fires by using spiking rule *a*^3^/*a*^2^ → *a*^2^, emitting 2 spikes to neuron *σ*_2_. In this way, the number of spikes in neuron *σ*_2_ becomes 5(*y* − 1) + 3 + 2 = 5*y*, which indicates number *y* is stored in register 2 of 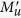.

The deterministic ADD module shown in Fig. 11 and SUB module shown in Fig. 4 can be used to simulate ADD and SUB instructions of 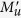. The FIN module shown in Fig. 7 can be used to output the computation result with changing neuron *σ*_1_ into *σ*_8_.

Until now, we have used

- 9 neurons for 9 registers,
- 25 neurons for 25 labels,
- 20 neurons for 10 ADD instructions,
- 28 neurons for 14 SUB instruction,
- 5 additional neurons in the INPUT module,

which comes to a total of 87 neurons.

This concludes the proof.

## Discussion and Future Works

In this work, a novel variant of SN P systems, namely SN P systems with self-organization, is introduced. As results, it is proven that the systems are Turing universal, i.e., they can compute and accept the family of sets of Turing computable natural numbers. With 87 neurons, the system can compute any Turing computable recursive function, thus achieving Turing universality.

There has been a research focus on the construction of small universal SN P with less computing resource, i.e. less number of neuron in use^17^,^48^,^49^,^50^,^51^,^52^. It is of interest that whether we can reduce the number of neurons in universal SN P systems with self-organization as function computing devices. A possible way is to construct ADD-ADD, ADD-SUB and SUB-ADD modules to perform particular consecutive ADD-ADD, ADD-SUB, and SUB-ADD instructions of 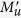.

SN P systems with learning function/capabiliy is a promising direction. Learning strategies and feedback mechanism have been intensively studied and investigated in conventional artificial neural networks. It is worthy to look into these techniques and transplant these ideas into SN P systems with self-organization.

In research of using artificial neural networks to recognize digital English letters, database MNIST (Mixed National Institute of Standards and Technology database) is widely used for training various letter recognition systems^53^, and for training and testing in the field of machine learning^54^. For further research, SN P systems with self-organization may be used to recognize handwritten digits letters and other possible pattern recognition problems. Since the data structure of SN P systems is binary sequences, an extra task of transmitting letters or pictures into binary sequences should be addressed. A possible way is transmitting digital numbers of pixels of pictures to binary form. Also, local binary pattern method, can be used to transmit pictures to binary forms.

Bioinformatics is s an interdisciplinary field that develops methods and software tools for understanding biological data^55^. Artificial intelligence based methods and data mining strategy have been used in processing biological data, see e.g.^56^,^57^,^58^,^59^,^60^,^61^,^62^, it is worthy to processing biological data by SN P systems, such as DNA motif finding^63^,^64^, nuclear export signal identification^65^,^66^.

## Additional Information

**How to cite this article**: Wang, X. *et al*. On the Computational Power of Spiking Neural P Systems with Self-Organization. *Sci. Rep.* **6**, 27624; doi: 10.1038/srep27624 (2016).
